# Introduction to an Open Source Internet-Based Testing Program for Medical Student Examinations

**DOI:** 10.3352/jeehp.2009.6.4

**Published:** 2009-12-20

**Authors:** Yoon-Hwan Lee

**Affiliations:** Department of Information and Statistics, Hallym University, Chuncheon, Korea.

**Keywords:** Internet-Based Testing, Computer-Based Testing, Medical Examination, Open Source Program

## Abstract

The author developed a freely available open source internet-based testing program for medical examination. PHP and Java script were used as the programming language and postgreSQL as the database management system on an Apache web server and Linux operating system. The system approach was that a super user inputs the items, each school administrator inputs the examinees' information, and examinees access the system. The examinee's score is displayed immediately after examination with item analysis. The set-up of the system beginning with installation is described. This may help medical professors to easily adopt an internet-based testing system for medical education.

## INTRODUCTION

Computer-based testing/computerized adaptive testing (CBT/CAT) has been used in many medical schools for the evaluation of students' achievement. CBT/CAT has also been applied to high stakes examinations such as the United States Medical Licensing Examination in the US. [1] and the Medical Council of Canada Qualifying Examination in Canada [2]. Recently, the National Health Personnel Licensing Examination Board, Republic of Korea, organized a task force on CBT/CAT. Although it is still not certain, CBT/CAT may be introduced into the Korean Medical Licensing Examination in the near future.

Internet-based testing (iBT) is another form of CBT/CAT based on the internet. Nowadays, most CBT/CAT is executed through iBT. This paper introduces an internet-based CBT program freely available to help medical professors implement iBT.

## MATERIALS AND METHODS

The iBT program is based on classical test theory unlike CAT based on item response theory. The operating system is Linux Fedora Core, the Web server is Apache 2.0x, which supports openSSL. The files used for iBT are http-2.24-2.1. fc6.rpm, openssl-0.9.8b-14.fc6 and openssl-devel-0.9.8b-14.fc6. The programming languages are PHP, of which version 5.0 or over is recommended. Files used are php-5.1.6-3.6.fc6.rpm, php-common-5.1.6-3.6.fc6, php-pgsql-5.1.6-3.6.fc6, and php-gd-5.1.6-3.6.fc6. The database management system is postgreSQL, of which version 8.0 or over is recommended. Files used are postgresql-8.1.9-1.fc6, postgresql-docs-8.1.9-1.fc6, postgresql-contrib-8.1.9-1.fc6, postgresql-libs-8.1.9-1.fc6, and postgresql-server-8.1.9-1.fc6. The goal is a straightforward interface between the administrator and examinees.

## RESULTS

The source file is available from sourceforge.net [3].

Installation

address: http://server_name/wbt
source directory: /usr/local/wbt

### Move to the directory to where the compressed source file will be uncompressed and uncompress the source file. Commands are as follows:

$ tar zxvf wbt_1_0.tar.gz
After the uncompression of the source file, the following four directories will appear.
  - data: Examination information is saved here. Web server can read and write.
  - html: Files accessible from web are allocated here.
  - include: Files necessary for site administration and configuration files are allocated here. Web server can read and write.
  - sqls: Table information is located here.

### Configuration of Apache web server

Access from the web server should be restricted only to the html directory. During rpm installation, *"httpd.conf"* is located in *"/etc/httpd/conf/."* The configuration file "httpd. conf" should be changed as follows and the rebooting of the web server is required.

Alias /wbt/ "/usr/local/wbt/html/"
Alias /wbt "/usr/local/wbt/html/"
<*Directory "/usr/local/wbt/html/"*>
  Options FollowSymLinks
  AllowOverride None
  Order allow,deny
  Allow from all
<*/Directory*>

### Change of permissions

Input the following commands in the prompt line. Even if this is determined during compression, it should be checked to prevent any unexpected problems.

$ chmod 707 /usr/local/wbt/data/exam
$ chmod 707 /usr/local/wbt/include
$ chmod 707 /usr/local/wbt/html/images/swf
$ chmod 707 /usr/local/wbt/html/images/testimages

### Execution of configuration file -revision of permissions

**Fig. 1:**
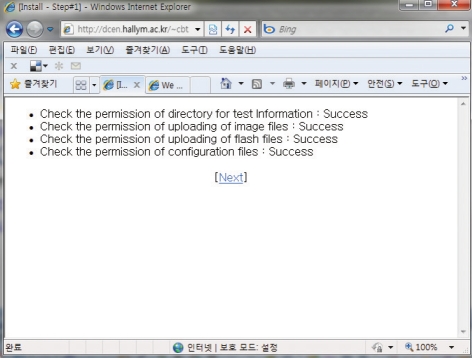
Screenshot seen if properpy executed during configuration of open source internet-based testing program.

**Fig. 2:**
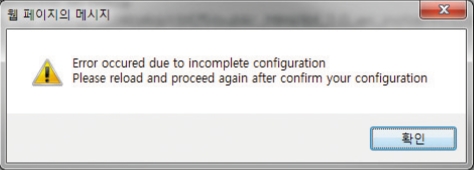
Error message seen due to incomplete configuration of open source internet-based testing program.

**Fig. 3:**
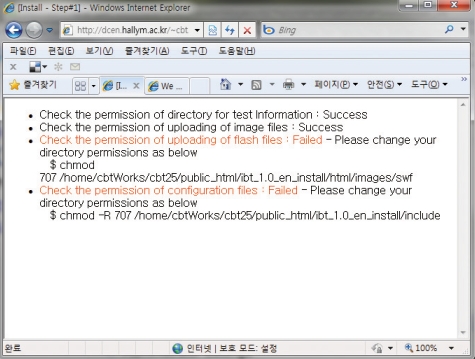
Solutions suggested to fix the error of configuration of open source internet-based testing program.

In the URL address window, input http://server_name/wbt/setup.php. If properly executed, the following screen shall appear (Fig. 1). Here, please put the Next icon so that the next screen appears. If there was no change of permissions in the previous procedure, the following screen will appear (Fig. 2) and the solution is provided in a guide on the screen (Fig. 3). Following the suggested solution, change the permissions and click O.K.

### Execution of the configuration file

**Fig. 4:**
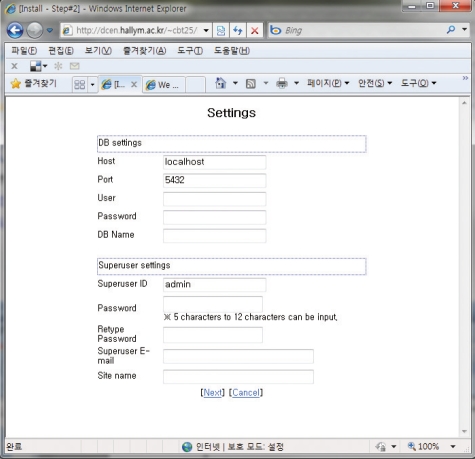
Form format for superuser's information of configuration of open source internet-based testing program.

**Fig. 5:**
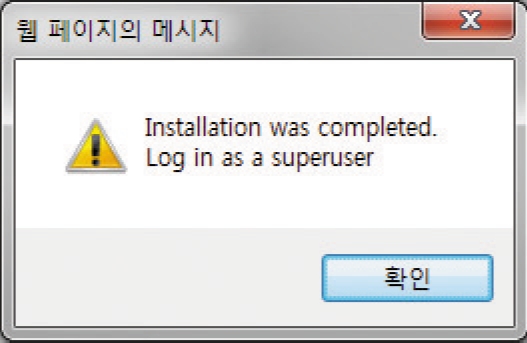
Screenshot after completing installation of configuration of open source internet-based testing program.

Input of the information for server and the super user: Here, server information and the superuser's information shall be inputted. Just input the data in the form format (Fig. 4). PostgreSQL's Port shall be determined. At this point only Port 5432 is supported. After inputting the information, click Next, so that Fig. 5. will appear. After that, the first screen will appear.

### Input of item data

The superuser can input the data.

### Input of the examinees' information

Each school's administrator can input the examinees' information.

## DISCUSSION

This installation procedure was presented in a workshop for CBT administration organized by the Korean Institute of Medical Education and Evaluation in December 5, 2009. Every participant can now install the iBT program on their web server. Therefore, any faculty members who have an interest in iBT will be able to install the program after downloading the source file from sourceforege.net. Since this program has already been used successfully at the Hallym University College of Medicine for more than 4 yr, it is possible to say the system is stable. Besides the merits of iBT over paper and pencil testing, this system displays items in random order and a screen with the answered and unanswered items marked to the side of the item. This allows examinees to postpone choosing an answer if an item is too difficult. The item analysis and the examinee's score can be obtained immediately after testing. Sill, several improvements are possible. First, the search and retrieval of items in the database would be helpful. Also, the item analysis according to item response theory would be an improvement. Although there are a few weaknesses of this program, it is already a powerful enough tool to perform iBT in medical schools. The maximum number of examinees has not yet been simulated but it should be possible for more than 1,000 students to participate in simultaneously.
